# P-1356. Risk factors associated with community-onset infections due to clinically moderate to high-risk AmpC producing organisms

**DOI:** 10.1093/ofid/ofae631.1533

**Published:** 2025-01-29

**Authors:** Nadeem Baalbaki, Mahinaz Mohsen, Moayad Al Hadidi, Leigh Ann Boyle, Joachim Sackey, Tanzila Salim

**Affiliations:** University Hospital, Newark, New Jersey; Rutgers New Jersey Medical School, Newark, New Jersey; Rutgers New Jersey Medical School, Newark, New Jersey; Rutgers Robert Wood Johnson Medical School, Newark, New Jersey; Rutgers School of Public Health, Newark, NJ; NJMS RUTGERS, Newark, New Jersey

## Abstract

**Background:**

The Enterobacterales order is among the most common group of bacteria relevant in clinical practice. Among them are inducible AmpC-producing Enterobacterales (AE). This unique group of bacteria may produce AmpC enzymes if induced in the setting of certain beta-lactam antibiotics, limiting the number of effective treatment options. Previous data have suggested that inappropriate antibiotics used empirically for AE increase the risk of treatment failure, but limited data are available to describe the risk factors associated with community-onset (CA) AE to help guide empiric therapy.

**Figure:**
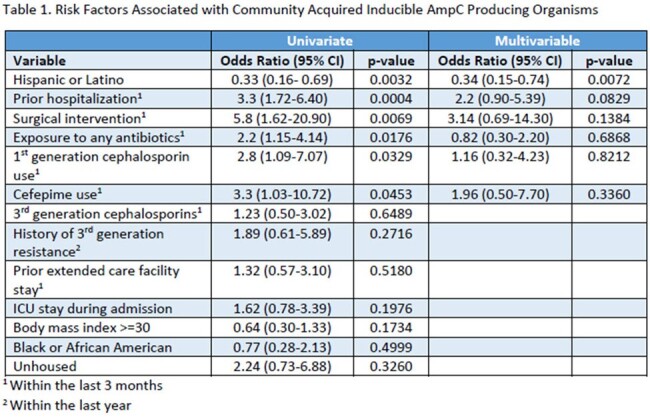

**Methods:**

This was a retrospective case-control study of patients admitted to University Hospital from 2021 to 2023 with a CA *Enterobacter cloacae*, *Klebsiella aerogenes*, or *Citrobacter freundii* clinical isolate. The first clinical isolate was included for each patient. Patients were matched at a rate of 1:1 to a patient with a CA Enterobacterales organism without 3rd generation cephalosporin resistance. Patients were matched according to infection, age (+/- 5 years), and sex at birth. The primary outcome was to evaluate risk factors associated with CA AE infections.

**Results:**

A total of 176 patients were included in this study, 88 in each group. In a univariate analysis, prior hospitalization (OR, 3.3 [95% CI, 1.72-6.4]), surgical intervention (OR, 5.8 [95% CI, 1.62-20.9]), exposure to any antibiotic (OR, 2.2 [95% CI, 1.15-4.14]), exposure to 1st generation cephalosporins (OR, 2.8 [95% CI, 1.09-7.07]), and exposure to cefepime (OR, 3.3 [95% CI, 1.03-10.72]) within 3 months were associated with an increased risk of a CA AE. Being Hispanic or Latino was associated with a decreased risk of having a CA AE in a univariate analysis and was the only variable statistically significant in a multivariable analysis (OR, 0.34 [95% CI, 0.15-0.74]).

**Conclusion:**

Our findings suggest that patients with prior hospitalization, surgical intervention, exposure to any antibiotic, to 1st generation cephalosporins, and to cefepime within 3 months may be associated with an increased risk of a CA AE in a univariate analysis, although no association was seen in a multivariable analysis. In contrast, Hispanic or Latino patients may be at a decreased risk for CA AE. A larger sample size may be warranted to validate these findings.

**Disclosures:**

**All Authors**: No reported disclosures

